# *Pratylenchus penetrans* Parasitizing Potato Crops: Morphometric and Genetic Variability of Portuguese Isolates

**DOI:** 10.3390/plants10030603

**Published:** 2021-03-23

**Authors:** Diogo Gil, Joana M.S. Cardoso, Isabel Abrantes, Ivânia Esteves

**Affiliations:** Centre of Functional Ecology, Department of Life Sciences, University of Coimbra, Calçada Martim de Freitas, 3000-456 Coimbra, Portugal; diogo.gil12@gmail.com (D.G.); joana.cardoso@uc.pt (J.M.S.C.); isabel.abrantes@uc.pt (I.A.)

**Keywords:** COI, cloning, ITS, morphometrics, plant-parasitic nematode, potato, molecular diversity

## Abstract

The root lesion *Pratylenchus penetrans* is an economically important pest affecting a wide range of plants. The morphometry of five *P. penetrans* isolates, parasitizing potato roots in Portugal, was compared and variability within and between isolates was observed. Of the 15 characters assessed, vulva position (V%) in females and the stylet length in both females/males showed the lowest coefficient of intra and inter-isolate variability. Moreover, DNA sequencing of the internal transcribed spacers (ITS) genomic region and cytochrome c oxidase subunit 1 (COI) gene was performed, in order to evaluate the intraspecific genetic variability of this species. ITS revealed higher isolate genetic diversity than the COI gene, with 15 and 7 different haplotypes from the 15 ITS and 14 COI sequences, respectively. Intra- and inter-isolate genetic diversity was found considering both genomic regions. The differentiation of these isolates was not related with their geographical origin. In spite of the high intraspecific variability, phylogenetic analyses revealed that both ITS region and COI gene separate *P. penetrans* from other related species. Our findings contribute to increasing the understanding of *P. penetrans* variability.

## 1. Introduction

The root lesion nematode (RLN) *Pratylenchus penetrans* (Cobb, 1917) Filipjev and Schuurmans Stekhoven, 1941 is an important migratory endoparasite, often reported as a limiting factor of several herbaceous and fruit crops [1,2,3]. On potato (*Solanum tuberosum* L.), the nematode causes necrotic lesions on tubers and roots due to migration and feeding, and its presence increases the severity of the “potato early dying” disease caused by *Verticillium dahliae* Kleb. [3,4]. Damage of roots by *P. penetrans* diminishes water and uptake of nutrients, resulting in poor plant growth and consequent crop losses. In Europe and North America, *P. penetrans* has been considered a damaging species associated to potato crop [5,6,7,8,9,10,11]. In Portugal, *P. crenatus* Loof, 1960, *P. neglectus* Filipjev and S. Stekhoven, 1941, *P. penetrans* and *P. thornei* Sher and Allen, 1953 have been found parasitizing potato, coexisting frequently in soil with other plant–parasitic nematodes [12]. The correct identification and characterization of *Pratylenchus* species are thus important, for example, to inform and advise farmers on the application of suitable pest management strategies. *Pratylenchus* species can be identified by means of morphological and morphometrical characters but requires specialized expertise since a considerable number of species share many morphological features and most specific differences can only be observed using high magnifications [3,13]. In addition, intraspecific morphological variability has been demonstrated in *P. penetrans* isolates in populations from different geographical locations [14]. To overcome the issues of overlapping morphological and morphometrical characters, identification of RLN should be complemented with the molecular analysis for accurate diagnosis of this group of nematodes [15]. Molecular methods based on restriction fragment length polymorphism (RFLP) analysis of the ribosomal ribonucleic acid (rRNA) genes [16] and sequencing of different fragments of the rDNA cluster, including internal transcribed spacers (ITS) [17,18], 18S [19,20] and 26S [19,21,22] have been used for diagnostics of RLN species [15]. Moreover, sequencing of the mitochondrial DNA (mtDNA), cytochrome c oxidase subunit 1 (COI) gene [15,23,24,25,26,27] and the nuclear hsp90 gene [23,24,27,28,29] have also been largely used in the molecular characterization of RLN species. *Pratylenchus fallax* Seinhorst, 1968, and *P. convallariae* Seinhorst, 1959, were shown to be closely related to *P. penetrans* (96–97% similarity) after sequence analysis of the D2–D3 region of the 28S rRNA gene [22,30]. Phylogenetic analyses of sequences of D2-D3 of 28S rDNA or partial 18S rDNA conducted by Subbotin et al. [19] grouped *P. penetrans* with *P. arlingtoni* Carta and Skantar, 2001, *P. convallariae*, *P. dunensis* de la Pena, van Aelst, Karssen and Moens, 2006, *P. fallax* and *P. pinguicaudatus* Corbett, 1969, in clade IV, forming the Penetrans group. Later, Palomares-Rius et al. [23] added *P. brachyurus* Filipjev and Schuurmans Stekhoven, 1941, and *P. oleae* n. sp. into this clade. Using a combination of phylogenetic data with molecular species delineation analysis, population genetics, morphometric information and sequences, Janssen et al. [15] reconstructed a multi-gene phylogeny of the Penetrans group using the ITS, D2-D3 of the 28S rDNA regions from nuclear rDNA and the COI gene from mtDNA. The authors were able to confirm the taxonomic status of *P. penetrans*, *P. fallax* and *P. convallariae*, clarifying the boundaries within the Penetrans group. In the same study, *P. fallax* populations demonstrated low intraspecific variability whereas *P. penetrans* showed diverse haplotypes, with extremely variable intraspecific variability. Nonetheless, identical *P. penetrans* haplotypes were found to be geographically widespread, suggesting that *P. penetrans* could have spread anthropogenically through agricultural development and crop exchange [15]. Despite *P. penetrans* has already been detected in Portuguese potato crops, little information is still available on the morphometric and molecular variability of these *P. penetrans* isolates. The knowledge acquired in this study can be valuable in help defining effective strategies for RLN management in this crop. Therefore, the objectives of this research were to evaluate the morphometric variability of *P. penetrans* Portuguese isolates and to assess their genetic diversity, geographical and host relations. Information on intra- and interspecific variation of *P. penetrans* parasitizing potato increase the awareness of the genetic diversity of this species, and relationships with other *P. penetrans* isolates in other countries and hosts.

## 2. Results

### 2.1. Morphology of P. penetrans Portuguese Isolates

#### 2.1.1. Female

**Figure 1:**
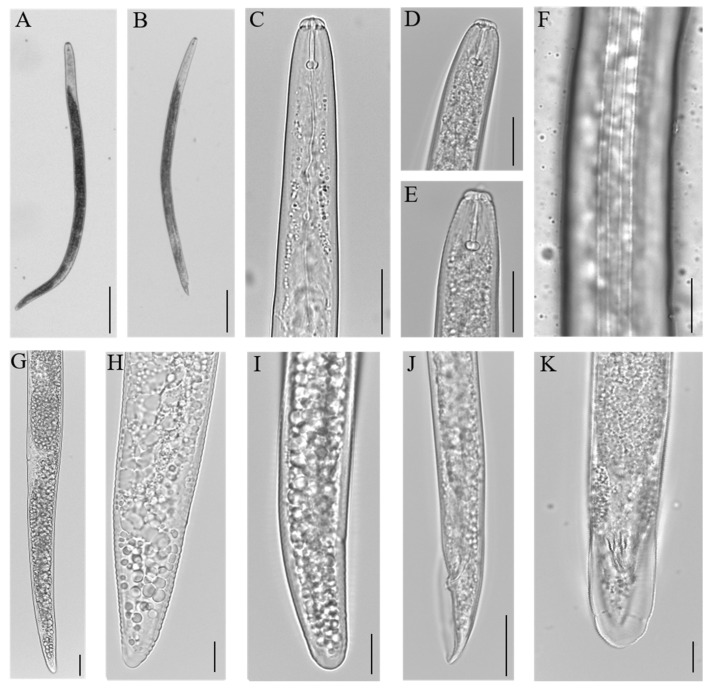
*Pratylenchus penetrans* specimens from Portuguese isolates: **A**—Entire female body, **B**—entire male body; **C**–**E**—anterior region; **F**—lateral field; **G**—female posterior region showing vulva; **H**,**I**—female tail variability; **J**—male posterior region; **K**—male tail (ventral side). Scale bars **A**,**B**: 100 μm; **C**–**E**, **J**: 20 μm; **F**–**I**, **K**:10 μm.

Body moderately slender, almost straight when heat relaxed, with body length 672.5 (522.1–869.5) μm long (Figure 1A and Table 1). Lip region slightly offset from body, body annules distinct, lip with three annules low flat anteriorly with rounded margins. Stylet stout 16.5 (15.2–17.9) μm long, with knobs varying from rounded to cupped anteriorly (Figure 1C–E). Lateral fields with four straight lines (Figure 1F). Pharyngeal glands overlapping intestine ventrally and slightly laterally. Excretory pore at from anterior extremity located opposite to pharyngo-intestinal junction. Spermatheca rounded, filled with sperm. Post uterine sac 1–1.5 times longer than vulval body diameter. Vulva located at 81.1 (75.7–83.9) % of body length (Figure 1G and Table 1). Tail cylindrical, 30.34 (19.0–41.5) μm long with smooth tip (Figure 1H,I and Table 1).

#### 2.1.2. Male

Males were common in all the isolates, morphologically similar to females for all non-sexual characters but smaller, with body length 555.64 (470.5–670.1) μm long (Figure 1B and Table 2). Lateral field with four lines ending on bursa, spicules slender, gubernaculum ventrally curved. Bursa irregularly crenate along margin, enveloping the tail tip (Figure 1J,K).

### 2.2. Morphometry of P. penetrans Portuguese Isolates

The morphometric measurements of *P. penetrans* isolates of Portugal were, in average, within the range described by Loof after [31], except for the c ratio in both PpA34L3 females and males, overall body length of PpA24L1 and PpA44L2 males and spicule length of PpA34L3 and PpA44L2 (Table 1 and Table 2). Morphometric comparisons using ANOVA revealed a significant degree of intra- and inter-isolate variability on most studied characters. Nine out of fifteen morphometric characters studied in *P. penetrans* females, varied significantly among isolates (*p* < 0.05) (Table 1). Inter-isolate variability was high for the overall body length, anterior end to excretory pore, anterior end to vulva, body width at anus, vulva–anus distance, tail length and ratios b’, c and c’, whereas the stylet length, distance of anterior end to median bulb, anterior end to pharyngeal gland lobe, maximum body width, V% and ratio a did not vary significantly among isolates (*p* > 0.05). The stylet length and V% had the lowest CV intra and inter-isolates of females and the highest values of CV were found in tail length, vulva–anus distance and b’ ratio (Table 3). In males, inter-isolate variability was found in nine out of thirteen morphometric characters: overall body length, anterior end to median bulb, anterior end to excretory pore, spicule length, tail and a, b’, c and c’ ratios (*p* < 0.05). The stylet length, distance from the anterior end to the tip of esophageal glands, body width at the anus and maximum body width were similar among isolates (*p* > 0.05) (Table 2). The stylet was the least variable character, whereas tail, c’ ratio and spicule length were the most variable among isolates, supporting the results given by the ANOVA (Table 4).

### 2.3. Genetic Diversity of P. penetrans Portuguese Isolates

ITS sequences of three clones from each isolate were determined and submitted in GenBank database under the accession numbers MW633839-MW633853. For the COI gene, sequences of two clones from isolate PpA21L2 and three clones from the other isolates were determined and also submitted under the accession numbers MW660605-MW660618. A BLAST search against NCBI database of the determined ITS and COI sequences confirmed the species identity, with sequences homologies ranging from 94.7% to 98.4%, and 99.2% to 100.00%, to other *P. penetrans* ITS and COI sequences, respectively, present in the database. The length variation on ITS region of all clones (671–683 bp) and the sequence analysis revealed high variability, not only between isolates but also within isolates, with a high number of polymorphic (S), mutation (Eta) sites and average number of nucleotide differences (k) then the ones found for COI region. All 15 ITS sequences and 14 COI sequences corresponded, respectively, to 15 and 7 different haplotypes (Table 5). Intra-isolate nucleotide diversity (Pi) for the ITS region was lower for the PpA34L3 isolate (Pi = 0.00997) and higher for the PpA44L2 isolate (Pi = 0.06115). For the COI gene, a low number of polymorphic and mutation sites were found considering each isolate or even considering all isolates. The COI intra-isolate Pi was lower for PpA44L2 isolate (Pi = 0.00000), with all three clones being identical, and higher for PpA21L2 isolate (Pi = 0.00509). Considering all isolates, a higher Pi was found for the ITS region (Pi = 0.03350) than for the COI gene (Pi = 0.00587) (Table 5).

### 2.4. Phylogenetic and Molecular Evolution Relationships

**Figure 2:**
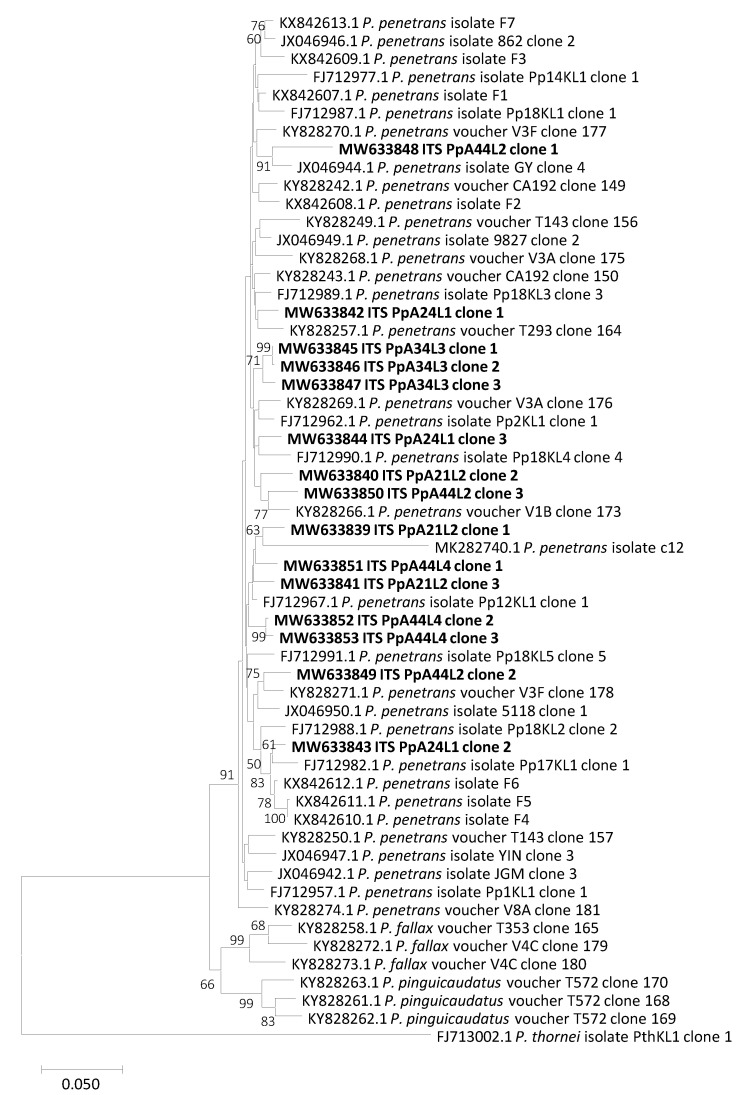
Neighbor-joining phylogenetic tree based on ITS nucleotide sequences of *Pratylenchus penetrans*, *P. pinguicaudatus* and *P. fallax*. ITS sequence from *P. thornei* was used as an outgroup. Bootstrap values are shown next to the branches and values with less than 50% confidence were not shown. Scale bar represents nucleotide substitutions per site.

**Figure 3:**
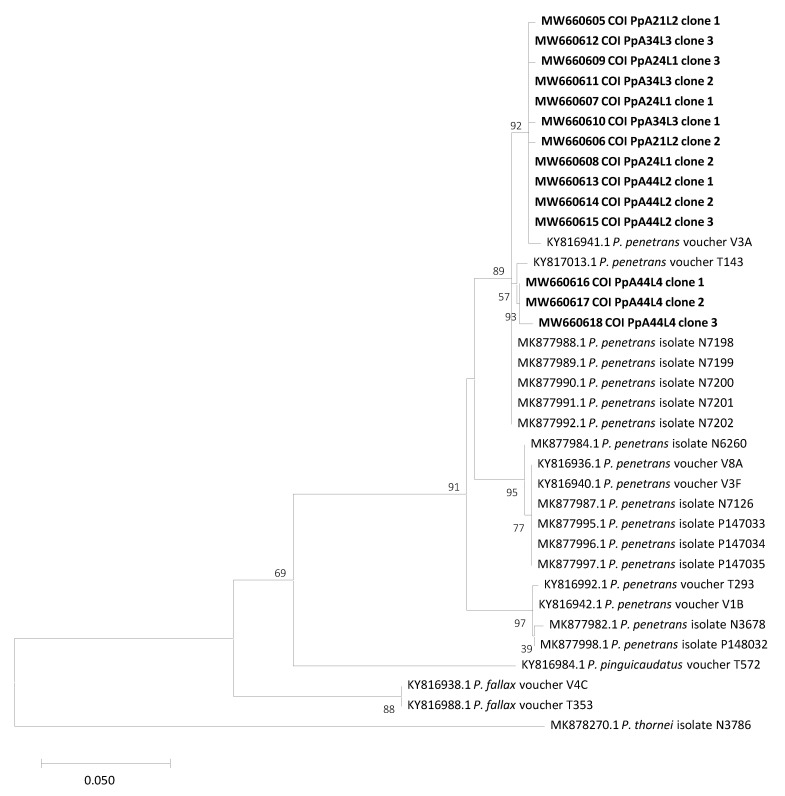
Neighbor-joining phylogenetic tree based on COI nucleotide sequences of *Pratylenchus penetrans*, *P. pinguicaudatus* and *P. fallax*. COI sequence from *P. thornei* was used as the outgroup. Bootstrap values are shown next to the branches and values with less than 50% confidence are not shown. Scale bar represents nucleotide substitutions per site.

Phylogenetic analysis was performed with the alignment of the 15 sequences obtained in this study and other ITS sequences from *P. penetrans*, *P. fallax*, *P. pinguicaudatus* and *P. thornei* present in the GenBank database. Results showed that *P. penetrans* isolates from Portugal clearly group up with other *P. penetrans* isolates but ITS sequences from the same isolates do not group together, reflecting the high intra- and inter-isolate estimated ITS divergence. Additionally, no grouping of isolates belonging to the same country or originated from the same host was found (Figure 2). On the other hand, phylogenetic analysis based on COI sequences revealed lower divergence between sequences from the same isolate and also from different isolates, comparing to the ITS region phylogenetic analysis. All Portuguese *P. penetrans* COI sequences grouped together and with other *P. penetrans* isolates, revealing a closer relationship with one Dutch isolate from apple (KY816941), one African isolate from onion (KY817013) and five American isolates from potato (MK877988; MK877989; MK877990; MK877991 and MK877992) (Figure 3). The differences between the *P. pinguicaudatus*, *P. fallax* and *P. thornei*, included in the phylogenetic analysis, were visible on both trees, as they did not group together (Figure 2 and Figure 3).

The estimate of evolutionary divergence between sequences of *P. penetrans* Portuguese isolates showed that ITS region diverges by at least 0.01513 base substitutions per site (±0.00487), considering different isolates and that value decreases for 0.00149 (±0.00149), considering intra-isolate divergence (isolate PpA34L3). However, there were ITS sequences from clones from the same isolate with an estimated divergence higher than from distinct isolates. The higher value of base substitutions per site, 0.08121 (±0.01253), was found between the PpA44L2 isolate, clone 1, and PpA24L1 isolate, clone 2 (Table S1). On the other hand, the COI gene revealed much lower nucleotide divergence with a minimum of 0.00000 base substitutions per site (±0.00000), considering both, intra and inter-isolate divergence. A maximum of 0.01821 (±0.00667) base substitutions per base on the COI gene was found between PpA21L2 isolate, clone 1 and PpA44L4 isolate, clone 3 (Table S2).

**Figure 4:**
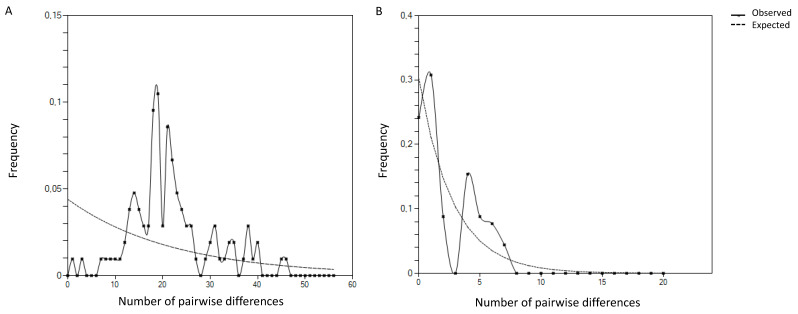
Mismatch distribution of ITS (**A**) and COI (**B**) sequences of five Portuguese isolates of *Pratylenchus penetrans*.

From neutrality tests, estimated Tajima’s D values, using the total number of mutations, were −1.54235 (*p* > 0.05) and −1.03620 (*p* > 0.05) for ITS and COI respectively, indicating that the changes were not significant and all sequences underwent neutral selection. Additionally, the mismatch distribution of both ITS and COI sequences revealed to be a multimodal distribution, with several peaks of pairwise differences, excluding the possibility of abrupt selection events (Figure 4).

The possible correlation of genetic distance and geographic distance of the five *P. penetrans* isolates were also investigated, considering both ITS and COI gene, and there were no significant correlation between this two variables with a Kendall tau of 0.02458 (*p* > 0.05) for ITS region and a Kendall tau of 0.08254 (*p* > 0.05) for the COI gene, showing that geographical distance is not the main factor leading to *P. penetrans* isolates differentiation.

## 3. Discussion

In this study, *P. penetrans* isolates from potato in different geographic locations of Portugal were characterized for the first time, using both morphometric and molecular analyses. The comparative morphometrical analyses revealed the presence of substantial inter and intra variability between isolates, although differences fall within the range of the morphometrical variability described previously in *P. penetrans* [3,31]. The body size of these isolates appears to be larger than that described by Rusinque et al. in *P. penetrans* parasitizing amaryllis (*Hippeastrum × hybridum*), in Portugal [32]. Spicule size of males and overall body length of the Portuguese isolates were also greater than those observed by Mokrini et al. in populations associated to maize (*Zea mays* L.) in Morocco [33]. Variations in morphometric characters can be caused from differences in fixation methods or changes in environmental conditions [34]. The morphometric characters of Portuguese isolates were recorded on fresh mounted nematodes (not glycerin-infiltrated specimens) and compared with type specimens in permanent mounts, and therefore affected by “shrinkage” due to the fixation process. Environmental conditions, like the host plant, influence morphometric characters such as body length, width, esophagus length, stylet length, V value, a and b ratios and qualitative characters such as tail terminus, growth of ovary and shape of the median bulb [14]. Townshend [35] reported that morphometric variations existed between populations of *P. penetrans* associated with strawberry (*Fragaria × ananassa*) and those associated with celery (*Apium graveolens* L.) in Ontario, Canada. Furthermore, variations in size were also found between *P. penetrans* populations recovered from strawberry collected at different geographical areas [35]. In our study, intra- and inter-isolate variability was found in most of the morphometric characters that were analyzed in females and males. However, the results obtained with ANOVA and the analysis of the CV allowed one to verify that the characters V and stylet length proved to be stable among isolates and between replicates within the same isolate. As previously noted by Roman and Hirschmann [13] and Tarte and Mai [14], the stability of these characters confirms its usefulness for discriminating this species. All other morphometrical characters, including those commonly used in nematode taxonomy (body length, body width, anterior end to esophageal glands and a, b’, c and c’ ratios), have shown relatively high coefficients of variation.

The ITS and COI genomic regions from the five Portuguese *P. penetrans* isolates were selected for sequencing to evaluate the intraspecific genetic diversity of this species. From the two regions, the ITS region revealed higher genetic diversity than the COI gene with 15 and 6 different haplotypes from the 15 ITS and 14 COI sequences, respectively. Besides, inter-isolate genetic diversity also intra-isolate genetic diversity was found in all isolates with exception for one isolate in the COI gene. Sequence comparisons performed by De Luca et al. [17] revealed high intraspecific variability in ITS sequences of several *Pratylenchus* species, including *P. penetrans*. Sequence analyses showed high sequence variability not only between populations or isolates but also within individuals. The same study concluded that ITS sequences allow a clear separation of the *Pratylenchus* species, despite the high intraspecific variability. Janssen et al. [15] also reported intraspecific variability of *P. penetrans* isolates based on sequence analysis and phylogenetic reconstruction of the ITS, D2-D3 regions of 28S rDNA and the COI gene. Furthermore, the phylogenetic analyses based on the sequences of the ITS and D2-D3 regions also confirmed high sequence variability among populations of *P. penetrans* [29].

Despite the high intraspecific diversity found for *P. penetrans* in our studies, phylogenetic analyses revealed that both ITS and COI genomic regions separate *P. penetrans* from other related species, such as *P. pinguicaudatus*, *P. fallax* and *P. thornei*, which is also in accordance with that previously reported [15,17,29]. Additionally, no grouping of isolates belonging to the same country or originated from the same host was found in phylogenetic analyses of both ITS and COI genomic regions. This is in agreement with the no correlation of genetic and geographic distance found among the Portuguese isolates, being the same COI haplotypes from isolates sampled in fields that are more than 90 km away, suggesting that geographical distance is not the main factor leading to the differentiation of isolates. Janssen et al. [15] referred that although the large intraspecific variability recovered in *P. penetrans*, identical haplotypes were found to be geographically widespread and this could be a result of the anthropogenic spread of *P. penetrans* through agriculture development and crop exchange. Our findings contribute to increase the understanding of *P. penetrans* variability.

## 4. Materials and Methods

### 4.1. Pratylenchus penetrans Isolates

Five *P. penetrans* isolates, obtained previously from potato roots sampled in the north and centre regions of mainland Portugal [12], were used in this study. The isolates were originated from a gravid female and propagated on carrot discs [36]. Isolates PpA21L2, PpA4L1 and PpA34L3 are from potato fields in different geographical locations, whereas PpA44L2 and PpA44L4 shared the same sampling geographic origin (Table 6).

### 4.2. Morphometrical Analyses

Twenty individual adults (10 females and 10 males), from each isolate, were mounted into a drop of water and used for the morphometric analyses. Before covering and sealing slides with the coverslips, nematodes were immobilized by gently heating the slide underneath, just enough to stop movement. Nematode measurements were made directly using a DM2500 microscope equipped with a ICC50HD digital camera (Leica Microsystems, Wetzlar, Germany) and LAS 4.8.0 software (Leica) and results compared with previous descriptions for this species [31]. Microscopic observations were made in nematodes without using a fixation method since the software used for nematode measurements allows the capture of the image and simultaneous measurement of specimens, without the need of a preservation method. All measurements were expressed in micrometers (μm). To assess the morphometric variation of the isolates, data was subjected to a one-way analysis of variance (ANOVA) using Statistica^®^ V.7 (StatSoft, Tulsa, Germany), after ensuring that the assumptions of normality and constant variance were met, as checked by using the Shapiro–Wilk and Levene’s tests, respectively. Logarithmic and square root transformations were applied to data whenever needed. Following ANOVA, to test differences between isolates Fisher Least Significant Difference test at the 95% confidence level was applied. The coefficients of variability (CV) were calculated to determine which characters were most stable and more variable among isolates.

### 4.3. DNA Extraction, PCR, Cloning and Sequencing

Nematode DNA was extracted from 50 to 100 mix developmental stages of *P. penetrans* PpA21L2, PpA24L1, PpA34L3, PpA44L2 and PpA44L4 isolates (Table 6) using the DNeasy^®®^ Blood and Tissue Mini kit (Qiagen, Hilden, Germany) following the manufacturer’s instructions.

Two genomic regions were selected to evaluate the intraspecific genetic diversity of this species, the internal transcribed spacers (ITS) rDNA region containing partial 18S and 28S and complete ITS1, 5.8S and ITS2 sequences and partial cytochrome c oxidase subunit I (COI) gene.

PCR amplifications were carried out using 20–50 ng extracted DNA and 2 U of BioTaq DNA polymerase (Meridian Bioscience, Memphis, TN, USA) in the 1× reaction buffer, 0.2 mM each dNTPs, 1.25 mM MgCl_2_ and 2.0 µM of each primer, PRATTW81 (5′GTAGGTGAACCTGCTGCTG3′) and AB28 (5′ATATGCTTAAGTTCAGCGGGT3′) for ITS region [16] and JB3 (5′TTTTTTGGGCATCCTGAGGTTTAT3′) and JB4.5 (5′TAAAGAAAGAACATAATGAAAATG3′) for the COI gene [37]. Reactions were carried out in a Thermal Cycler (Bio-Rad, California, USA) with an initial denaturation step of 95 °C for 3 min followed by 35 reaction cycles of 94 °C for 30 s, annealing for 30 s at 60 °C and 54 °C for ITS region and COI region, respectively, extension at 72 °C for 30 s and a final extension at 72 °C for 7 min. The PCR products were purified using the NucleoSpin^®®^ Gel and PCR Clean-up kit (Macherey-Nagel, Duren, Germany) according to the manufacturer’s instructions and cloned. Purified ITS and COI amplified products were ligated into pGEM^®®^-T Easy Vector (Promega, Madison, USA using 50 ng vector in a 10 µL reaction with 3 U T4 DNA Ligase (Promega) and 36 ng purified ITS or 22 ng of COI products in the 1× Rapid Ligation Buffer (Promega). Ligation reactions were incubated for 1 h at room temperature. Then, 2 µL of the ligation product was used to transform *Escherichia coli* JM109 high efficiency competent cells (Promega) following the manufacturer’s instructions. Plasmid DNA was extracted from *E. coli* cells using the Nzymini Prep kit (Nzytech, Lisbon, Portugal and three selected positive clones for each genomic region and each *P. penetrans* isolate were fully sequenced in both strands in an Automatic Sequencer 3730xl under BigDyeTM terminator cycling conditions at Macrogen Company (Madrid, Spain).

### 4.4. Sequence Analysis

Sequence analysis and alignments were achieved using BioEdit [38]. The region containing primers sequence was removed from all sequence analyses. Homologous sequences in the databases were searched using the Basic Local Alignment Search Tool [39]. Sequence statistics such as number of polymorphic (S) and mutation (Eta) sites, nucleotide diversity (Pi), haplotype diversity (Hd), average number of nucleotide differences (k) and mismatch distributions were estimated using DnaSP 6.12.03 software [40]. Intra-isolate sequence analyses were performed from the alignments obtained with sequences of each isolate and overall sequence diversity with the alignment obtained with all sequences of the five isolates.

### 4.5. Phylogenetic and Molecular Evolutionary Analyses

Phylogenetic and molecular evolutionary analyses were conducted in MEGA v10.1.8 software [41]. Phylogenetic trees were constructed by the neighbor-joining method [42] with 1000 replications of bootstrap, with the evolutionary distances computed using the maximum composite likelihood [43] model and ambiguous positions removed for each sequence pair (pairwise deletion option), using the ITS and COI nucleotide sequence alignments of the five isolates used in this study and homologous sequences retrieved from the GenBank database (Table 7). Genetic distance between sequences from the five Portuguese isolates were accomplished by pairwise distance using the maximum composite likelihood model with pairwise deletion option and standard error estimate by a bootstrap procedure (1000 replicates), using the alignments of ITS and COI nucleotide sequences determined in this study. Additionally, Tajima’s D neutrality tests [44], which distinguish between a DNA sequence evolving randomly (or neutrally) and one evolving under a non-random process, and mismatch distribution of ITS and COI sequences of Portuguese *P. penetrans* isolates were performed in DnaSP v6.12.03 software.

The correlation between genetic and geographic distance of Portuguese *P. penetrans* isolates was also evaluated computing the determined pairwise distance versus the distance between the sampling locations of each of the five isolates. Geographic distance between isolates was calculated using the script available at https://www.movable-type.co.uk/scripts/latlong.html (accessed on 15 January 2021) with the GPS coordinates of each isolate sampling location (Table 6 and Table S3). The significance of genetic and geographic distance correlation was calculated using Kendall tau rank correlation in Free Statistics Software v1.2.1 [45].

## 5. Conclusions

In conclusion, morphometric and genetic diversity were found among *P. penetrans* isolates and this variability was not only a result of the diversity found between isolates but also due to the diversity within each isolate. The information gathered highlights the importance of the knowledge about this relevant plant–parasitic nematode in potato crops, and can be used further in larger genetic studies, focusing this nematode species. Future research should also be conducted to evaluate whether the differences in pathogenicity among *P. penetrans* isolates are related to the observed morphometric and molecular variability.

## Figures and Tables

**Table 1:** Morphometric characters of *Pratylenchus penetrans* females from Portugal. All measurements are in μm. Data are means of 10 nematodes ± standard deviation (range). In each row means followed by the same letters do not differ significantly at *p* > 0.05, according to the Fisher Least Significant Difference test.

Character	Isolate					Loof (1960)
	PpA21L2	PpA24L1	PpA34L3	PpA44L2	PpA44L4	
L *	630.3 ± 59.4 ^a,b,c^ (527.5–720.0)	723.5 ± 93.9 ^c,d^ (603.3–858.8)	600.8 ± 86.3 ^a,b^ (522.1–812.1)	712.2 ± 61.0 ^c,d^ (615.5–802.5)	695.6 ± 82.4 ^b,c,d^ (605.3–869.5)	343–811
Stylet length	16.9 ± 0.8 ^a^ (16.0–17.5)	16.2 ± 0.6 ^a^ (15.4–17.2)	16.9 ± 1.2 ^a^ (16.7–17.9)	16.2 ± 0.6 ^a^ (15.2–17.3)	16.5 ± 0.7 ^a^ (15.9–17.6)	15–17
Anterior end to medium bulb	57.2 ± 3.6 ^a^ (50.7–62.3)	63.5 ± 7.6 ^a^ (49.4–71.8)	61.8 ± 5.1 ^a^ (53.7–70.5)	61.9 ± 2.7 ^a^ (56.0–66.2)	60.9 ± 5.3 ^a^ (49.4–67.9)	
Anterior end to esophageal gland lobe	125.4 ± 15.6 ^a^ (103.4–147.7)	136.2 ± 15.3 ^a^ (108.4–156.7)	137.3 ± 10.5 ^a^ (126.2–155.2)	134.0 ± 9.2 ^a^ (120.7–151.9)	124.7 ± 21.7 ^a^ (82.4–148.0)	
Anterior end to excretory pore	89.0 ± 9.1 ^a,b^ (78.4–105.6)	98.7 ± 9.3 ^b,c,d^ (81.6–113.3)	91.4 ± 9.7 ^a,b,c^ (83.9–116.8)	104.7 ± 4.3 ^c,d^ (96.0–111.7)	92.9 ± 15.3 ^a,b,c^ (71.7–114.5)	
Anterior end to vulva	510.6 ± 45.5 ^a^ (423.7–575.9)	586.1 ± 77.5 ^a^ (485.6–695.8)	496.0 ± 77.8 ^a^ (427.0–697.0)	574.5 ± 53.9 ^a^ (474.2–637.5)	558.9 ± 77.6 ^a^ (475.9–724.8)	
Maximum body width	28.2 ± 5.1 ^a^ (21.9–39.2)	34.1 ± 6.7 ^a^ (23.5–43.8)	30.0 ± 3.7 ^a^ (23.6–34.2)	31.2 ± 4.5 ^a^ (23.2–36.4)	33.0 ± 5.9 ^a^ (24.2–43.4)	
Body width at anus	14.8 ± 1.7 ^a,b^ (11.6–17.3)	17.5 ± 2.5 ^b,c,d^ (13.2–21.0)	15.6 ± 1.8 ^a,b,c^ (13.5–19.2)	18.2 ± 2.8 ^c,d^ (13.2–24.0)	16.9 ± 2.3b,^c,d^ (13.9–20.2)	
Vulva-anus	87.6 ± 16.4 ^a,b,c^ (64.9–118.7)	103.5 ± 17.2 ^c,d^ (72.7–127.7)	79.6 ± 14.6 ^a,b^ (58.9–111.0)	104.6 ± 18.0 ^c,d^ (80.9–141.7)	94.1 ± 11.3 ^b,c,d^ (72.9–109.0)	
Tail	29.4 ± 4.9 ^a,b^ (19.0–35.8)	31.8 ± 2.6 ^a,b,c^ (27.7–35.6)	23.7 ± 3.9 ^d^ (19.5–31.4)	34.0 ± 4.2 ^b,c^ (26.6–41.0)	32.8 ± 3.9 ^a,b,c^ (25.9–38.4)	
V *	81.1 ± 2.2 ^a^ (75.7–83.7)	81.0 ± 1.7 ^a^ (77.7–83.9)	82.5 ± 2.2 ^a^ (78.3–83.9)	80.6 ± 2.0 ^a^ (77.0–83.5)	80.3 ± 4.0 ^a^ (79.8–83.4)	75–84
a *	22.8 ± 3.5 ^a^ (17.4–28.3)	21.6 ± 3.0 ^a^ (18.5–26.9)	20.1 ± 2.3 ^a^ (16.1–23.8)	23.1 ± 3.1 ^a^ (20.1–29.3)	21.4 ± 2.4 ^a^ (16.5–25.0)	19–32
b’ *	5.1 ± 0.6 ^a,b,c^ (4.3–6.4)	5.3 ± 0.5 ^c^ (4.8–6.3)	4.4 ± 0.4 ^a^ (3.8–5.2)	5.3 ± 0.5 ^c^ (4.6–6.0)	5.9 ± 2.0 ^c^ (4.3–10.5)	
c *	21.9 ± 3.3 ^a^ (18.1–29.6)	22.9 ± 3.2 ^a^ (17.6–27.2)	25.6 ± 2.8 ^b^ (21.1–28.9)	21.2 ± 2.3 ^a^ (18.5–25.6)	21.3 ± 2.3 ^a^ (18.2–24.6)	15–24
c’ *	2.0 ± 0.3 ^a^ (1.4–2.5)	1.9 ± 0.4 ^a^ (1.5–2.7)	1.5 ± 0.3 ^b^ (1.2–1.9)	1.9 ± 0.4 ^a^ (1.4–2.4)	2.0 ± 0.3 ^a^ (1.5–2.4)	

* L—body length; V—position of vulva from the anterior end expressed as the percentage of body length; a—body length/maximum body width; b’—body length/distance from the anterior end to the base of esophageal glands; c—body length/tail length; c’—tail length/tail diameter at the anus.

**Table 2:** Morphometric characters of *Pratylenchus penetrans* males from Portugal. All measurements are in μm. Data are means of 10 nematodes ± standard deviation (range). In each row, means followed by the same letters do not differ significantly at *p* > 0.05, according to the Fisher Least Significant Difference test.

Character	Isolate					Loof (1960)
	PpA21L2	PpA24L1	PpA34L3	PpA44L2	PpA44L4	
L *	535.0 ± 30.7 ^a^ (483.3–582.4)	582.6 ± 35.0 ^b^ (529.0–629.4)	522.0 ± 32.2 ^a^ (470.5–580.5)	602.6 ± 36.0 ^b^ (531.5–670.1)	536.0 ± 17.9 ^a^ (508.2–570.0)	305–574
Stylet length	15.8 ± 0.4 ^a^ (15.3–16.7)	15.3 ± 0.4 ^a^ (14.9–15.7)	15.7 ± 0.6 ^a^ (15.0–16.5)	15.6 ± 0.6 ^a^ (14.8–16.7)	15.9 ± 1.1 ^a^ (14.4–17.5)	
Anterior end to medium bulb	57.5 ± 3.1 ^a,b^ (52.7–62.9)	53.6 ± 4.6 ^b,c^ (45.7–59.2)	59.5 ± 3.4 ^a,b^ (54.6–63.7)	63.7 ± 4.4 ^d^ (54.9–71.5)	56.0 ± 3.9 ^a,b,c^ (49.5–60-5)	
Anterior end to esophageal gland lobe	124.0 ± 12.9 ^a^ (105.2–149.2)	122.3 ± 6.6 ^a^ (110.9–131.2)	122.5 ± 8.0 ^a^ (110.7–137.9)	127.7 ± 10.2 ^a^ (108.5–144.0)	127.9 ± 9.5 ^a^ (112.0–146.2)	
Anterior end to excretory pore	85.0 ± 5.0 ^a^ (79.0–92.6)	83.2 ± 6.9 ^a^ (71.7–94.2)	84.5 ± 7.7 ^a^ (72.3–97.6)	94.9 ± 6.0 ^b^ (81.7–104.1)	87.1 ± 5.7 ^a^ (77.0–95.9)	
Maximum body width	21.9 ± 2.4 ^a^ (18.2–27.1)	20.8 ± 1.7 ^a^ (17.7–23.5)	21.3 ± 1.8 ^a^ (19.1–25.0)	21.2 ± 1.7 ^a^ (19.2–24.4)	19.8 ± 4.3 ^a^ (8.6–23.2)	
Body width at anus	14.0 ± 1.4 ^a^ (12.0–15.7)	13.3 ± 0.7 ^a^ (12.4–14.6)	13.3 ± 0.7 ^a^ (12.2–14.9)	14.3 ± 1.2 ^a^ (12.3–15.8)	13.8 ± 0.7 ^a^ (12.9–14.8)	
Spicule	16.2 ± 0.8 ^a^ (15.2–17.5)	15.8 ± 1.5 ^a^ (13.8–18.0)	19.1 ± 1.6 ^b^ (16.6–21.6)	18.6 ± 1.7 ^b^ (15.9–21.9)	16.7 ± 1.4 ^a^ (14.4–18.5)	14–17
Tail	25.6 ± 2.9 ^a,b^ (22.4–32.7)	29.0 ± 4.1 ^b,c^ (21.7–35.4)	21.9 ± 2.6 ^d^ (16.1–24.9)	28.2 ± 3.8 ^a,b,c^ (22.5–36.0)	27.4 ± 2.8 ^a,b,c^ (23.6–31.8)	
a *	24.6 ± 2.5 ^a^ (19.8–29.4)	28.2 ± 2.6 ^b^ (25.3–34.4)	24.6 ± 2.4 ^a^ (19.9–27.1)	28.5 ± 1.6 ^b^ (26.8–30.5)	29.3 ± 11.9 ^b^ (22.8–62.6)	23–34
b’ *	4.4 ± 0.5 ^a^ (3.4–4.9)	4.8 ± 0.4 ^b^ (4.2–5.5)	4.3 ± 0.2 ^a^ (4.0–4.6)	4.7 ± 0.2 ^b^ (4.4–5.1)	4.2 ± 0.4 ^a^ (3.7–4.8)	
c *	21.1 ± 2.5 ^a^ (16.4–24.3)	20.4 ± 2.5 ^a^ (15.6–24.4)	24.2 ± 3.4 ^b^ (20.7–31.4)	21.6 ± 1.9 ^a^ (18.6–24.1)	19.7 ± 1.9 ^a^ (16.9–22.8)	16–22
c’ *	1.8 ± 0.2 ^a,b^ (1.6–2.2)	2.2 ± 0.4 ^b,c^ (1.5–2.7)	1.6 ± 0.2 ^a^ (1.2–2.0)	2.0 ± 0.3 ^a,b,c^ (1.5–2.4)	2.0 ± 0.3 ^a,b,c^ (1.7–2.5)	

* L—body length; V—position of vulva from anterior end expressed as percentage of body length; a—body length/maximum body width; b’—body length/distance from anterior end to base of esophageal glands; c—body length/tail length; c’—tail length/tail diameter at anus.

**Table 3:** Intra- and inter-isolate coefficient of variability (%) of *Pratylenchus penetrans* females from Portugal.

Character	Isolate					Inter-Isolate Coefficient of Variability (%)
	PpA21L2	PpA24L1	PpA34L3	PpA44L2	PpA44L4	
L *	9.4	13.0	14.4	8.6	11.8	8.0
Stylet length	4.6	3.9	7.2	3.9	4.5	2.2
Anterior end to medium bulb	6.4	11.9	8.2	4.4	8.7	3.8
Anterior end to esophageal gland lobe	12.5	11.2	7.6	6.9	17.4	4.6
Anterior end to excretory pore	10.3	9.5	10.6	4.1	16.5	6.6
Anterior end to vulva	8.9	13.2	15.7	9.4	13.9	7.3
Maximum body width	18.2	19.8	12.4	14.4	17.9	7.5
Body width at anus	11.2	14.5	11.3	15.3	13.4	8.4
Vulva-anus	18.7	16.6	18.3	17.2	12.1	11.3
Tail	16.6	8.3	16.5	12.4	12.0	13.5
V *	2.8	2.0	2.7	2.5	5.0	1.0
a *	15.3	13.8	11.3	13.2	11.4	5.5
b’ *	12.7	8.5	9.1	9.6	33.5	10.5
c *	14.9	13.9	10.8	10.9	10.6	8.1
c’ *	16.2	19.2	17.1	19.2	14.3	10.1

* L—body length; V—position of vulva from anterior end expressed as percentage of body length; a—body length/maximum body width; b’—body length/distance from anterior end to base of esophageal glands; c—body length/tail length; c’—tail length/tail diameter at the anus.

**Table 4:** Intra- and inter-isolate coefficient of variability (%) of *Pratylenchus penetrans* males from Portugal.

Character	Isolate					Inter-Isolate Coefficient of Variability (%)
	PpA21L2	PpA24L1	PpA34L3	PpA44L2	PpA44L4	
L *	5.7	6.0	6.2	6.0	3.3	6.3
Stylet length	2.7	2.8	3.6	3.9	6.7	1.4
Anterior end to medium bulb	5.4	8.5	5.7	6.9	7.0	6.6
Anterior end to esophageal gland lobe	10.4	5.4	6.5	8.0	7.5	2.2
Anterior end to excretory pore	5.8	8.3	9.1	6.3	6.5	5.4
Maximum body width	11.0	8.1	8.3	8.1	21.7	3.7
Body width at anus	9.9	5.6	5.3	8.4	5.0	3.3
Spicule	4.9	9.3	8.3	8.9	8.4	8.7
Tail	11.3	14.2	12.0	13.4	10.2	10.7
a *	10.3	9.2	9.6	5.6	40.7	8.3
b’ *	10.7	8.1	4.5	4.4	8.7	5.9
c *	11.6	12.2	13.9	8.9	9.6	8.0
c’ *	10.2	16.4	13.3	13.6	12.5	10.5

* L—body length; V—position of vulva from anterior end expressed as percentage of body length; a—body length/maximum body width; b’—body length/distance from anterior end to base of esophageal glands; c—body length/tail length; c’—tail length/tail diameter at the anus.

**Table 5:** Genetic diversity of cloned ITS and COI regions of five *Pratylenchus penetrans* isolates from Portugal.

Isolate	Genomic Region	No. of Clones	Sequences Length (bp)	S *	Eta *	No. of Haplotypes	Hd * (Standard Deviation)	Pi * (Standard Deviation)	K *
**PpA21L2**	ITS	3	677; 683; 673	35	35	3	1.000 (0.272)	0.03488 (0.00254)	23.333
	COI	2	393	2	2	2	1.000 (0.500)	0.00509 (0.00964)	2.000
**PpA24L1**	ITS	3	676; 673; 671	38	42	3	1.000 (0.272)	0.03992 (0.01097)	26.667
	COI	3	393	1	1	2	0.667 (0.314)	0.00170 (0.00080)	0.667
**PpA34L3**	ITS	3	671; 671; 673	10	10	3	0.667 (0.314)	0.00997 (0.00425)	6.667
	COI	3	393	1	1	2	1.000 (0.272)	0.00170 (0.00080)	0.667
**PpA44L2**	ITS	3	673; 675; 678	60	62	3	1.000 (0.272)	0.06115 (0.01759)	40.667
	COI	3	393	0	0	1	0.000 (0.000)	0.0000 (0.0000)	0.000
**PpA44L4**	ITS	3	676; 674; 674	20	20	3	1.000 (0.272)	0.00339 (0.00160)	13.333
	COI	3	393	2	2	2	0.667 (0.314)	0.01990 (0.00765)	1.333
**All 5 isolates**	ITS	15	-	99	109	15	1.000 (0.024)	0.03350 (0.00414)	21.743
	COI	14	-	10	10	7	0.758 (0.116)	0.00587 (0.00164)	2.308

* S—number of polymorphic sites; Eta—total number of mutations; Hd—haplotype diversity; Pi—nucleotide diversity; k—average number of nucleotide differences.

**Table 6:** *Pratylenchus penetrans* isolates used in this study, respective geographical origin and GenBank accession numbers.

Isolate	GPS Coordinates	Locality	Accession (ITS)	Accession (COI)
PpA21L2	41°16′18″ N 8°41′23″ W	Aveleda, Maia, Portugal	MW633839 MW633840 MW633841	MW660605 MW660606 -
PpA24L1	41°15′27″ N 8°40′30″ W	Vila Nova da Telha, Maia, Portugal	MW633842 MW633843 MW633844	MW660607 MW660608 MW660609
PpA34L3	40°37′28″ N 8°38′19″ W	Aveiro, Portugal	MW633845 MW633846 MW633847	MW660610 MW660611 MW660612
PpA44L2	40°23′25.2″ N 8°30′07.7″ W	Coimbra, Portugal	MW633848 MW633849 MW633850	MW660613 MW660614 MW660615
PpA44L4	40°23′25.2″ N 8°30′07.7″ W	Coimbra, Portugal	MW633851 MW633852 MW633853	MW660616 MW660617 MW660618

**Table 7:** Sequences used in this study.

Species	Isolate	Region	Host	Acession Number	
				ITS	COI
*Pratylenchus fallax*	T353	The Netherlands, Doornenburg	*Malus pumila*	KY828258	KY816988
*P. fallax*	V4 C	The Netherlands, Ysbrechtum	*Vitis vinifera*	KY828272;KY828273	KY816938
*P. penetrans*	V3 A	The Netherlands, Baarlo	*M. pumila*	KY828268;KY828269	KY816941
*P. penetrans*	V8 A	The Netherlands, Baarlo	*M. pumila*	KY828274	KY816936
*P. penetrans*	V1B	The Netherlands, Meijel	*M. pumila*	KY828266	KY816942
*P. penetrans*	V3 F	The Netherlands, Nagele	*M. pumila*	KY828270; KY828271	KY816940
*P. penetrans*	N3678	USA, Minnesota	*Zea mays*	-	MK877982
*P. penetrans*	N6260	USA, Fairbanks County	*Paeonia* sp.	-	MK877984
*P. penetrans*	N7126	USA, Otoe County	*Malus* sp.	-	MK877987
*P. penetrans*	N7198	USA, Idaho	*Solanum tuberosum*	-	MK877988
*P. penetrans*	N7199	USA, Idaho	*S. tuberosum*	-	MK877989
*P. penetrans*	N7200	USA, Idaho	*S. tuberosum*	-	MK877990
*P. penetrans*	N7201	USA, Idaho	*S. tuberosum*	-	MK877991
*P. penetrans*	N7202	USA, Idaho	*S. tuberosum*	-	MK877992
*P. penetrans*	P148032	USA, Portage County	*S. tuberosum*	-	MK877998
*P. penetrans*	P147033	USA, Portage County	*S. tuberosum*	-	MK877995
*P. penetrans*	P147034	USA, Portage County	*S. tuberosum*	-	MK877996
*P. penetrans*	P147035	USA, Portage County	*S. tuberosum*	-	MK877997
*P. penetrans*	c12	Canada, Kentville	*Prunus* sp.	MK282740	-
*P. penetrans*	862	Chile	*Lillium* sp.	JX046946	-
*P. penetrans*	GY	France	*Prunus* sp.	JX046944	-
*P. penetrans*	JGM	France	*Sambucus* sp.	JX046942	-
*P. penetrans*	CA192	France, Britany	*M. pumila*	KY828242;KY828243	-
*P. penetrans*	Pp18KL1	Long Island, USA	*S. tuberosum*	FJ712987	-
*P. penetrans*	Pp18KL2	Long Island, USA	*S. tuberosum*	FJ712988	-
*P. penetrans*	Pp18KL3	Long Island, USA	*S. tuberosum*	FJ712989	-
*P. penetrans*	Pp18KL4	Long Island, USA	*S. tuberosum*	FJ712990	-
*P. penetrans*	Pp18KL5	Long Island, USA	*S. tuberosum*	FJ712991	-
*P. penetrans*	F1	MN, USA	*S. tuberosum*	KX842607	-
*P. penetrans*	F2	MN, USA	*S. tuberosum*	KX842608	-
*P. penetrans*	F3	MN, USA	*S. tuberosum*	KX842609	-
*P. penetrans*	F4	MN, USA	*S. tuberosum*	KX842610	-
*P. penetrans*	F5	MN, USA	*S. tuberosum*	KX842611	-
*P. penetrans*	F6	MN, USA	*S. tuberosum*	KX842612	-
*P. penetrans*	F7	MN, USA	*S. tuberosum*	KX842613	-
*P. penetrans*	Pp17KL1	Monroe County, USA	*Prunus cerasus*	FJ712982	-
*P. penetrans*	Pp12KL1	Rennes, France	*Malus* sp.	FJ712967	-
*P. penetrans*	T143	Rwanda, Nyakiriba	*Allium. cepa*	KY828249;KY828250	KY817013
*P. penetrans*	Pp14KL1	Spain	*Malus* sp.	FJ712977	-
*P. penetrans*	9827	The Netherlands	*Iris* sp.	JX046949	-
*P. penetrans*	5118	The Netherlands	*Lillium* sp.	JX046950	-
*P. penetrans*	T293	The Netherlands, Apeldoorn	*Pyrus* sp.	KY828257	KY816992
*P. penetrans*	Pp1KL1	Tongeren, Belgium	*Rubus* sp.	FJ712957	-
*P. penetrans*	YIN	USA	*Acer x freemanii*	JX046947	-
*P. penetrans*	Pp2KL1	Zandhoven, Belgium	*Z. mays*	FJ712962	-
*P. pinguicaudatus*	T572	UK, England, Rothemstadt	*Triticum* sp.	KY828261;KY828262;KY828263	KY816984
*P. thornei*	N3786	California, USA	*V. vinifera*	-	MK878270
*P. thornei*	PthKL1	Santaella, Spain	*Cicer arietinum*	FJ713002	-

## Data Availability

The data presented in this study are available in Plants 2021, 10, 603. https://doi.org/10.3390/plants10030603. DNA sequence information were deposited in GenBank database under the accession numbers: MW633839-MW633853 (ITS sequences) e MW660605-MW660618 (COI sequences).

## Supplementary Materials

The following are available online at https://www.mdpi.com/2223-7747/10/3/603/s1, Table S1: Data on genetic distance among the 15 internal transcribed spacers (ITS) sequences from the five Portuguese *Pratylenchus penetrans* isolates; Table S2: Data on genetic distance among the 14 cytochrome c oxidase subunit 1 (COI) gene sequences from the five Portuguese *Pratylenchus penetrans* isolates; Table S3: Data on geographic distance among the five Portuguese *Pratylenchus penetrans* isolates. Geographic distance (Km) estimated by the distance between the GPS coordinates of each of the five isolates sampling locations.
