# Supplementary material for: Pratylenchus penetrans Parasitizing Potato Crops: Morphometric and Genetic Variability of Portuguese Isolates
Source: Plants (Basel). 2021 Mar 23;10(3):603. doi: 10.3390/plants10030603 (PMC8004771; doi:10.3390/plants10030603)
Supplement: Supplementary file 1 [file plants-10-00603-s001.pdf]

Supplementary Table S1. Data on genetic distance among the 15 internal transcribed spacers (ITS) sequences from the five Portuguese *Pratylenchus penetrans* isolates. Genetic distance estimated by the number of base substitutions per site (below the diagonal) and respective standard error (above the diagonal).

	PpA21L2_1	PpA21L2_2	PpA21L2_3	PpA24L1_1	PpA24L1_2	PpA24L1_3	PpA34L3_1	PpA34L3_2	PpA34L3_3	PpA44L2_1	PpA44L2_2	PpA44L2_3	PpA44L4_1	PpA44L4_2	PpA44L4_3
**PpA21L2_1**		0.00813	0.00781	0.00808	0.00991	0.00895	0.00765	0.00785	0.00771	0.01120	0.01025	0.00921	0.00834	0.00785	0.00816
**PpA21L2_2**	0.04144		0.00719	0.00783	0.00931	0.00743	0.00647	0.00658	0.00650	0.01092	0.00957	0.00834	0.00780	0.00694	0.00739
**PpA21L2_3**	0.03531	0.03367		0.00801	0.00810	0.00545	0.00467	0.00487	0.00574	0.00935	0.00790	0.00722	0.00557	0.00655	0.00700
**PpA24L1_1**	0.03817	0.03359	0.03844		0.00906	0.00830	0.00769	0.00788	0.00743	0.01092	0.00944	0.00897	0.00817	0.00776	0.00832
**PpA24L1_2**	0.05737	0.05116	0.04011	0.04760		0.00862	0.00771	0.00785	0.00760	0.01253	0.00690	0.00937	0.00774	0.00828	0.00871
**PpA24L1_3**	0.04344	0.03369	0.01826	0.03858	0.04349		0.00571	0.00594	0.00628	0.01005	0.00897	0.00792	0.00788	0.00792	0.00840
**PpA34L3_1**	0.03538	0.02594	0.01361	0.03372	0.03538	0.01982		0.00149	0.00450	0.01005	0.00772	0.00670	0.00633	0.00685	0.00720
**PpA34L3_2**	0.03695	0.02749	0.01513	0.03528	0.03694	0.02136	0.00149		0.00476	0.01022	0.00797	0.00687	0.00648	0.00703	0.00736
**PpA34L3_3**	0.03682	0.02747	0.02127	0.03205	0.03682	0.02289	0.01359	0.01511		0.00960	0.00815	0.00744	0.00665	0.00704	0.00742
**PpA44L2_1**	0.06623	0.06444	0.05007	0.06445	0.08121	0.05519	0.05671	0.05833	0.05332		0.01189	0.01165	0.01082	0.01095	0.01134
**PpA44L2_2**	0.06067	0.05257	0.03846	0.04912	0.02879	0.04340	0.03686	0.03842	0.03993	0.07271		0.00941	0.00835	0.00831	0.00873
**PpA44L2_3**	0.04495	0.04121	0.03067	0.04166	0.04812	0.03377	0.02603	0.02758	0.03219	0.06971	0.05110		0.00844	0.00789	0.00811
**PpA44L4_1**	0.03979	0.03821	0.02263	0.03812	0.03511	0.03836	0.02731	0.02885	0.03038	0.06418	0.04287	0.04149		0.00664	0.00709
**PpA44L4_2**	0.03504	0.03052	0.02751	0.03348	0.04153	0.03715	0.02917	0.03073	0.03066	0.06292	0.04146	0.03547	0.02737		0.00300
**PpA44L4_3**	0.03816	0.03364	0.03063	0.03660	0.04470	0.04033	0.03231	0.03387	0.03380	0.06621	0.04462	0.03706	0.03047	0.00596	

Supplementary Table S2. Data on genetic distance among the 14 cytochrome c oxidase subunit 1 (COI) gene sequences from the five Portuguese *Pratylenchus penetrans* isolates. Genetic distance estimated by the number of base substitutions per site (below the diagonal) and respective standard error (above the diagonal).

	**PpA21L2_1**	**PpA21L2_2**	**PpA24L1_1**	**PpA24L1_2**	**PpA24L1_3**	**PpA34L3_1**	**PpA34L3_2**	**PpA34L3_3**	**PpA44L2_1**	**PpA44L2_2**	**PpA44L2_3**	**PpA44L4_1**	**PpA44L4_2**	**PpA44L4_3**
**PpA21L2_1**		0.00340	0.00237	0.00237	0.00345	0.00364	0.00237	0.00237	0.00237	0.00237	0.00237	0.00566	0.00566	0.00667
**PpA21L2_2**	0.00514		0.00247	0.00247	0.00353	0.00361	0.00247	0.00247	0.00247	0.00247	0.00247	0.00564	0.00564	0.00657
**PpA24L1_1**	0.00256	0.00255		0.00000	0.00253	0.00265	0.00000	0.00000	0.00000	0.00000	0.00000	0.00513	0.00513	0.00615
**PpA24L1_2**	0.00256	0.00255	0.00000		0.00253	0.00265	0.00000	0.00000	0.00000	0.00000	0.00000	0.00513	0.00513	0.00615
**PpA24L1_3**	0.00513	0.00512	0.00255	0.00255		0.00372	0.00253	0.00253	0.00253	0.00253	0.00253	0.00573	0.00573	0.00662
**PpA34L3_1**	0.00514	0.00512	0.00255	0.00255	0.00512		0.00265	0.00265	0.00265	0.00265	0.00265	0.00592	0.00592	0.00683
**PpA34L3_2**	0.00256	0.00255	0.00000	0.00000	0.00255	0.00255		0.00000	0.00000	0.00000	0.00000	0.00513	0.00513	0.00615
**PpA34L3_3**	0.00256	0.00255	0.00000	0.00000	0.00255	0.00255	0.00000		0.00000	0.00000	0.00000	0.00513	0.00513	0.00615
**PpA44L2_1**	0.00256	0.00255	0.00000	0.00000	0.00255	0.00255	0.00000	0.00000		0.00000	0.00000	0.00513	0.00513	0.00615
**PpA44L2_2**	0.00256	0.00255	0.00000	0.00000	0.00255	0.00255	0.00000	0.00000	0.00000		0.00000	0.00513	0.00513	0.00615
**PpA44L2_3**	0.00256	0.00255	0.00000	0.00000	0.00255	0.00255	0.00000	0.00000	0.00000	0.00000		0.00513	0.00513	0.00615
**PpA44L4_1**	0.01293	0.01290	0.01029	0.01029	0.01289	0.01290	0.01029	0.01029	0.01029	0.01029	0.01029		0.00000	0.00351
**PpA44L4_2**	0.01293	0.01290	0.01029	0.01029	0.01289	0.01290	0.01029	0.01029	0.01029	0.01029	0.01029	0.00000		0.00351
**PpA44L4_3**	0.01821	0.01817	0.01552	0.01552	0.01815	0.01817	0.01552	0.01552	0.01552	0.01552	0.01552	0.00512	0.00512	

Supplementary Table S3. Data on geographic distance among the five Portuguese *Pratylenchus penetrans* isolates. Geographic distance (Km) estimated by the distance between the GPS coordinates of each of the five isolates sampling locations.

	PpA21L2_1	PpA21L2_2	PpA21L2_3	PpA24L1_1	PpA24L1_2	PpA24L1_3	PpA34L3_1	PpA34L3_2	PpA34L3_3	PpA44L2_1	PpA44L2_2	PpA44L2_3	PpA44L4_1	PpA44L4_2	PpA44L4_3
**PpA21L2_1**		0.000	0.000	1.999	1.999	1.999	72.100	72.100	72.100	99.260	99.260	99.260	99.260	99.260	99.260
**PpA21L2_2**			0.000	1.999	1.999	1.999	72.100	72.100	72.100	99.260	99.260	99.260	99.260	99.260	99.260
**PpA21L2_3**				1.999	1.999	1.999	72.100	72.100	72.100	99.260	99.260	99.260	99.260	99.260	99.260
**PpA24L1_1**					0.000	0.000	70.460	70.460	70.460	97.520	97.520	97.520	97.520	97.520	97.520
**PpA24L1_2**						0.000	70.460	70.460	70.460	97.520	97.520	97. 520	97.520	97.520	97.520
**PpA24L1_3**							70.460	70.460	70.460	97.520	97.520	97.520	97.520	97.520	97.520
**PpA34L3_1**								0.000	0.000	28.470	28.470	28.470	28.470	28.470	28.470
**PpA34L3_2**									0.000	28.470	28.470	28.470	28.470	28.470	28.470
**PpA34L3_3**										28.470	28.470	28.470	28.470	28.470	28.470
**PpA44L2_1**											0.000	0.000	0.000	0.000	0.000
**PpA44L2_2**												0.000	0.000	0.000	0.000
**PpA44L2_3**													0.000	0.000	0.000
**PpA44L4_1**														0.000	0.000
**PpA44L4_2**															0.000
**PpA44L4_3**															
